# Preparation and Evaluation of Ribonuclease-Resistant Viral HIV RNA Standards Based on Armored RNA Technology

**DOI:** 10.29252/.22.6.394

**Published:** 2018-11

**Authors:** Mohammad Gholami, Mehrdad Ravanshad, Kazem Baesi, Siamak M. Samiee, Negin Hosseini Rozbahani, Minoo Mohraz

**Affiliations:** 1 Department of Medical Virology, Faculty of Medical Sciences, Tarbiat Modares University, Tehran, Iran; 2 Hepatitis and AIDS Department, Pasteur Institute of Iran, Tehran, Iran; 3 Food and Drug Laboratory Research Center, Ministry of Health and Medical Education, Tehran, Iran; 4 Department of Immunology, Faculty of Medicine, Tehran University of Medical Sciences, Tehran, Iran; 5 Iranian Research Center for HIV AIDS (IRCHA), Iranian Institute for Reduction of High-Risk Behaviors, Tehran University of Medical Science, Tehran, Iran

**Keywords:** HIV-1, Real-time PCR, Virus-like particle

## Abstract

**Background:**

The human immunodeficiency virus type 1 (HIV-1) is an infectious viral agent that gradually extinguishes the immune system, resulting in acquired immune deficiency syndrome (AIDS). The aim of this study was to construct an RNA-positive control based on armored (AR) RNA technology, using HIV-1 RNA as a model.

**Methods:**

The MS2 maturase, a coat protein gene (at positions 1765 to 1787) and HIV-1 *pol* gene were cloned into pET-32a plasmid. The prepared plasmid was transformed into *Escherichia coli* strain BL2 (DE3), and the expression of the construct was induced by 1 mM of isopropyl-L-thio-D-galactopyranoside (IPTG) at 37 °C for 16 h to obtain the fabricated AR RNA. The AR RNA was precipitated and purified using polyethylene glycol and Sephacryl S-200 chromatography.

**Results:**

The stability of AR RNA was evaluated by treatment with DNase I and RNase A and confirmed by transmission electron microscopy and gel agarose electrophoresis. Tenfold serial dilution of AR RNA from 10^1^ to 10^5^ was prepared. Real-time PCR assays had a range of detection between 10^1^ and 10^5^. In addition, R^2^ value was 0.998, and the slope of the standard curve was -3.33.

**Conclusion:**

Prepared AR RNA, as a positive control, could be used as a basis for launching an in-house HIV-1 virus assay and other infectious agents. It can be readily available to laboratories and HIV research centers. The AR RNA is non-infectious and highly resistant to ribonuclease enzyme and can reduce the risk of infection in the clinical laboratory.

## INTRODUCTION

Human immunodeficiency virus (HIV) infection is one of the most critical challenges to global public health[1,2]. The accurate evaluation of HIV type 1 (HIV-1) RNA levels is the most important factor for understanding the natural history of HIV infection, monitoring the progression of the disease to acquired immune deficiency syndrome (AIDS) and determining the efficacy of antiretroviral therapies.[3,4].

PCR-based assays are common methods for the evaluation of viruses and the related transcripts in biological samples. However, controlling the reverse transcription step in PCR reaction and especially applying a control similar to the template sequence are critical requirements of this method.

Researchers have recently reported the encapsidation of target RNA in another viral capsid such as MS2, which improved the RNA stability[5]. The MS2 is a single-stranded RNA (+ssRNA) virus with positive-sense strand and icosahedral capsid, which belongs to the Leviviridae family. Its genomic size is nearly 3.6 kb, which encodes four proteins, including coat protein, maturase protein (A-protein), lysis protein, and replicase protein[6]. The MS2 attaches to the fertility factor (F) on the *E. coli* and enters the bacteria using maturase protein. After entering the bacteria, the viral RNA acts as messenger RNA and translates into structural proteins. In the MS2 assembling stage, one molecule of viral +ssRNA, one copy of the maturase protein (A-protein), and 180 copies of coat (14 kDa) protein are required[7].

The phage packaging starts by binding 180 copies of coat protein to a specific region in a hairpin- like structure consisting of 19 nucleotides, which is located near 5’ side of the phage genome. Among these nucleotides, residues A-4, U-5, and A-7 act as recognition sites in the hairpin and play a role in the phage packaging[7,8]. In the structure of MS2 capsid, maturase protein protects the phage particle from degradation by ribonuclease enzyme[9,10].

According to the “armored (AR) RNA’’ technology, almost any exogenous RNA in an appropriate size can be integrated into downstream of the Pac site of MS2 phage genome and non-specifically assembled into expressed MS2 phage capsid, leading to the formation of target AR RNA by phage-like particles (PLPs). Once quantified, the AR RNA can be used as substitute controls and standards for the clinical diagnosis of virus RNA by RT-PCR and qRT-PCR[11]. The aim of this study was to provide an insight into the production of ribonuclease-resistant AR RNA MS2 PLP using HIV-1 RNA as a model, based on AR RNA technology.

## MATERIALS AND METHODS

### Bacterial strains and vectors

*E. coli strain* BL21 (DE3) was used as the host strain for the expression of our target (MS2-HIV, MS2_-1751nt_ + HIV POL_336nt_). The pET32a plasmid containing ampicillin resistance gene and T7 promoter was used as expression vector in this study.

### MS2 and HIV *pol* sequence amplification

Commercial MS2 phage was purchased from Sigma-Aldrich (Germany). MS2 maturase, coat protein gene (1751 bp), and HIV-1 *pol* (336 bp) gene were amplified by specific primers (Table 1) using Superscript® III one-step RT-PCR System with Platinum® Taq High-Fidelity DNA Polymerase kit (Invitrogen, USA). Amplification of MS2 RNA was done at 45 °C for 1 h for the synthesis of cDNA. PCR was then performed at 94 °C for 2 min, followed by 38 cycles of denaturation at 94 °C for 30 s, annealing at 64 °C for 35 s, extension at 68 °C for 2 min, and a final extension temperature of 68 °C for 5 min. For HIV-1 *pol* gene amplification, the cDNA synthesis step was carried out at 45 °C for 30 min, and the amplification cycles were 95 °C for 2 min, 38 cycles of denaturation at 94 °C for 30 s, annealing at 64 °C for 35 s, extension at 68 °C for 40 min, and a final extension temperature of 68 °C for 5 min. As presented in Table 1, MS2 primers have a *Bam*HI and *Hind*III restriction site (underlined sequences), while the HIV-1 *pol* primer sequences contained *Hind*III and *Not*I restriction sites.

**Table 1:** Primers sequences used for PCR and real-time PCR amplification

Primer	Sequence
MS2-F	5’-ATGGATCCCCTTTCGGGGTCCTGCTC-3’
MS2-R	5’-GCAAGCTTAGTTGAACTTCTTTGTTGTCTTCG-3’
HIV-F	5’- GCAAGCTTTGAAAGAAAAGGGGGGATTGGG-3’
HIV-R	5’-AAAAGCGGCCGCGTT TTACTA AACTGT TCCATGT-3’
HIV-F	5’-GGCCAATGGACATATCAAATTTATC-3’
HIV-R	5’-CTGCCTCTGTTAATTGTTTTACATC-3’
Probe	5’-TTAGTGTGGGCACCCCTCATTCTTGC-3’

### Construction of recombinant *E. coli* strain BL21 (DE3)/pET32a-MS2

The amplified MS2 sequence was cleaned up and cloned into *Bam*HI/*Hind*III site of pET32a plasmid using standard protocols. The resulting plasmid, pET32a-MS2, was propagated in *E. coli* BL21 (DE3) and extracted with Qiagen miniprep plasmid extraction kit (Qiagen, Germany), as per the manufacturer’s instruction.

### Construction of recombinant *E. coli* strain BL21 (DE3)/pET32a-MS2-HIV

To generate the recombinant plasmid of pET32a-MS2-HIV, the HIV PCR product was first digested by *Hind*III (Fermentase, USA) and *Not*I (Takara, Japan) restriction enzymes. The digested product was then cleaned with Qiagen PCR clean-up kit and cloned into the *Hind*III/ *Not*I sites downstream of the MS2 pac site on pET32a. Each DNA fragment described above was verified by direct DNA sequencing before digestion with restriction enzyme and prior to insertion into linearized pET32a.

### Expression of pET32a-MS2-HIV

The *E. coli* strain BL21 (DE3) harboring pET32a-MS2-HIV was cultured in 1 L LB broth medium containing 50 μg/ml of ampicillin and grown in a shaking incubator at 200 ×g at 37 °C for 12 h. The protein expression was then induced by adding 1 mM of isopropyl-L-thio-D-galactopyranoside (IPTG) at the same conditions for 16 h. Subsequently, the bacterial pellet was collected by centrifugation and disrupted by sonication. The cell debris was removed by centrifugation at 16,000 ×g at 4 °C for 10 min. The supernatant contained AR RNA particles, *E. coli* genome, and DNA plasmid. RNA and DNA contaminations were removed by treatment with 10 mM CaCl_2_, RNase A, and DNase I (Thermo Fisher Scientific, USA).

### Precipitation of AR-MS2 + HIV-1

The supernatant was collected and transferred into a 50-ml conical tube, followed by the slow addition of NaCl up to a final concentration of 2%, with constant and gentle stirring. Next, polyethylene glycol (PEG) 6000 was added to a final concentration of 10%; the mixture was stirred for about 1 h to ensure complete solubilization of the PEG. The beaker was transferred to a refrigerator, and the virus and other proteins were allowed to precipitate at 4 °C for 72 h. The precipitate was collected by centrifuging at 15,000 ×g at 4 °C for 20 minutes. The precipitate was suspended in a small volume of TES buffer (0.01 M of Tris-HCI, pH 7.2, 0.002 M of EDTA, and 0.15 M of NaCl). Finally, the AR was purified by a Sephacryl S-200 resin in TES buffer (10 mM of Tris, pH 7.5, 1 mM of EDTA, and 100 mM of NaCl).

### Stability validation of AR-MS2 + HIV-1

The PLPs stability was analyzed by agarose gel electrophoresis (1%) and staining with gel red dye. The intact AR was confirmed by transmission electron microscopy (TEM), followed by negative staining with 1% phosphotungstic acid.

### Quantification of AR-MS2 + HIV-1

The number of MS2 PLP was determined by UV spectrophotometry using the Avogadro’s constant, extinction coefficient of 0.125 mg/ml of MS2 bacteriophage per absorbance unit at 1 OD 260 nm, and a molecular weight of 3.0 × 10^6^, as previously described [12].

### Primers and probes design and RT-PCR detection of AR-MS2 + HIV-1

The primers and probe were designed by AlelleID ver. 6 and Beacon Designer ver. 8 (PREMIER Biosoft, USA) and confirmed in the most conserved region of the *pol* gene of the HIV-1 genome. The latter sequence was downloaded from HIV database (https://www.hiv.lanl.gov/content/index), truncated and inserted at down-stream of MS2 genome. The primers and probe sequences, as shown in Table 1, were sent to the Metabion Company (Germany) for synthesis with reporter dye fluorescein amidite (FAM), 5(6)carboxy-fluorescein and the Black Hole Quencher dys, which were conjugated at the 5’ and 3’ ends. Tenfold serial dilutions containing 10^5^ to 10^1^ copies/µl of AR particles were assayed. To do so, RNA molecule was first released by incubation at 80 °C for 5 min. After that, real-time PCR detection was performed using step one real-time PCR instrument (Applied Biosystems, USA), as well as QuantiTect Probe RT-PCR Kit (Qiagen, Germany) and TaqMan Master Mix. Reaction was prepared and performed with 12.5 µl buffer, 300 nm forward and reverse primers, 150 nm probe, and 12 µl AR RNA. The RT reaction lasted for 35 min at 50 °C, followed by a denaturation step for 15 min at 95 °C and 12 cycles of 15 s at 95 °C, 35 s at 62 °C, and 35 s at 72 °C without fluorescent collection, followed by 40 cycles of 10 s at 94 °C, and annealing and extension for 40 s at 60 °C. The fluorescent signal was acquired at the annealing-extension step at 60 °C.

## RESULTS

### Agarose gel electrophoresis of HIV-1 (*pol*) and MS2 fragments

**Fig. 1:**
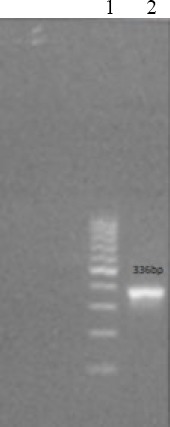
Results of RT-PCR and PCR with 1% agarose gel analysis. Lane 1, ladder 100 bp; lane 2, RT-PCR of HIV *pol* (336 bp).

**Fig. 2:**
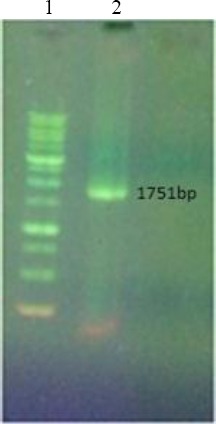
Results of RT-PCR and PCR with 1% agarose gel analysis. Lane 1, ladder 1 kb; lane 2, RT-PCR of MS2 (1751 bp).

Figures 1 and 2 illustrate the MS2 maturase, coat protein gene, and HIV-1 *pol* gene (336 bp), which were obtained by RT-PCR from MS2 RNA (Roche, Germany) using Superscript® III One-Step RT-PCR System with Platinum® Taq High-Fidelity DNA Polymerase kit (Invitrogen, USA).

### Transmission electron microscopy of AR-MS2 + HIV-1

**Fig. 3:**
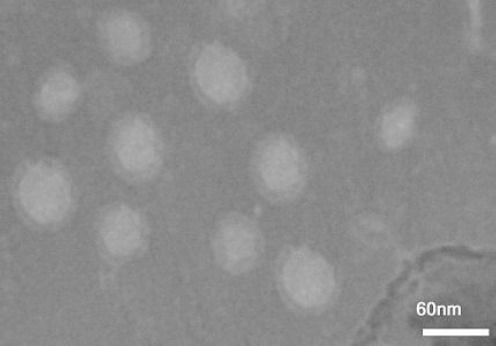
Transmission electron microscopy. Phage-like particles with approximately 30 nm in diameter were found clearly.

AR particles were precipitated with PEG 6000 and negatively stained with 1% phosphotungstic acid. The TEM image confirmed that AR particles had icosahedral shape and a 30-nm diameter (Fig. 3).

### Stability evaluation of expressed AR-MS2 + HIV-1

**Fig. 4:**
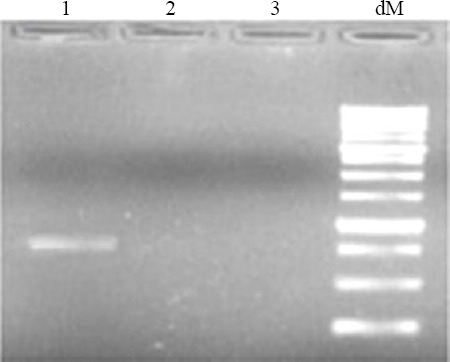
Resistance of armored (AR) RNA to RNase and DNase treatment. Lane 1, AR RNA HIV-1 after purification with Sephacryl S-200; lanes 2 and 3, unprotected RNA after digestion with DNase and RNase; lane 4, ladder 1 kB; dM, DNA marker.

**Fig 5:**
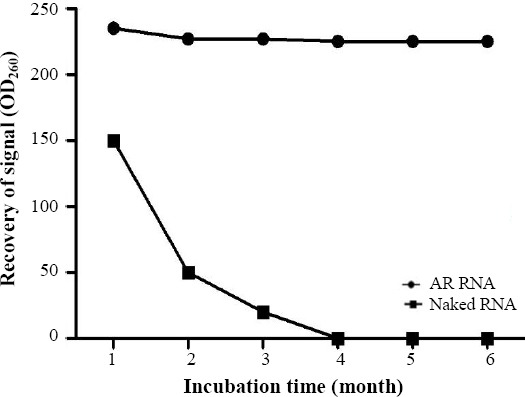
Comparison between the stability of armored (AR) RNA and naked RNA at -4 °C. The concentration of naked and AR RNA was estimated by measuring the OD absorbance at 260 nm (Thermo Scientific™ NanoDrop™). AR RNA was stable, but the naked RNA rapidly degraded.

The AR-MS2 + HIV-1 was completely resistant to DNase and RNase treatment after 4 h at 37 °C (Fig. 4), wherein the naked RNA was degraded rapidly. As previously described, AR-MS2 + HIV-1 particles were detected in a band, migrating at about 900–1,000 bp[13,14]. Moreover, the stability of naked and AR RNA was evaluated by incubating in 10 mM of Tris (pH 7.0), 100 mM of NaCl, and 1 mM of MgCl_2_ (TSM) at -4 °C over six months. The AR RNA was stable in +4 °C, although naked RNA was rapidly degraded (Fig. 5).

### SDS-PAGE analysis of AR-MS2 + HIV-1 product

**Fig. 6:**
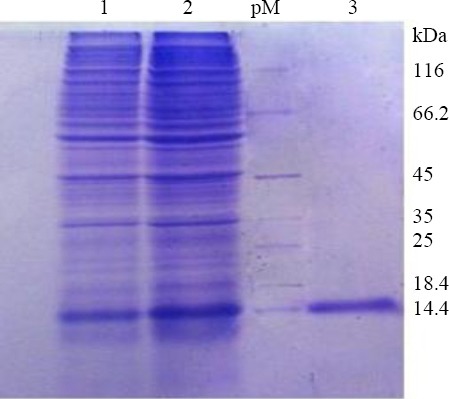
MS2 phage purification by Sephacryl S-200 chromatography. Lane 1, *E. coli* BL21, MS2-HIV (PEG precipitation); lane 2, MS2-HIV (ultra-sonication); lane 3, MS2-HIV after purification.; pM, protein marker

SDS-PAGE analysis of induced BL21 (DE3)/pET-32a MS2-HIV confirmed the expression of an ~14-kDa protein (Fig. 6). This 14-kDa expressed protein was very close to the reported size of 14 kDa capsid protein of the MS2 phage, which indicates the successful expression of designed MS2-HIV in *E. coli* BL21 (DE3).

### Real-time RT-PCR detection of AR-MS2 + HIV-1

**Fig. 7:**
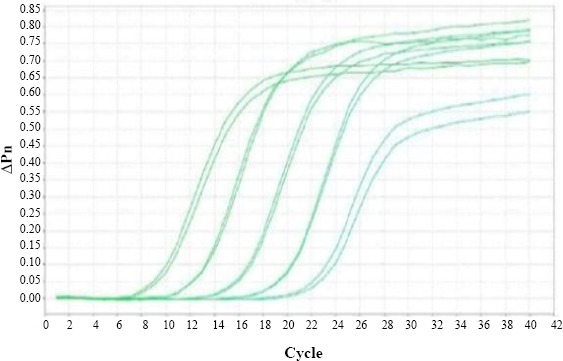
Amplification plot of HIV-1 *pol* gene in engineered armored RNA-MS2 phage (Serial dilution from 10^1^ to 10^5^) by real-time PCR.

A tenfold serial dilution of AR-MS2 + HIV-1 from 10^5^ to 10^1^ was prepared. The RT-PCR was first performed on standard to obtain the limit of detection (LoD) of the assay. As shown in Figure 7, this assay had a LoD varying between 1 and 10^5^ with R^2^ value and standard curve slope of 0.998 and -3.33, respectively, suggesting constantly high amplification efficiency (greater than 99%).

**Fig. 8:**
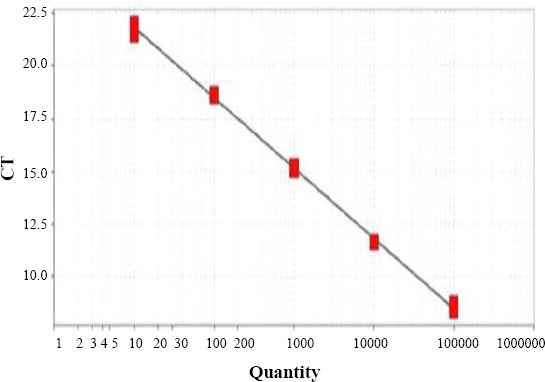
Standard curve from serial dilution (10^1^ to 10^5^) of HIV-1 *pol* gene inserted in armored MS-2 phage with slopes of -3.33, R^2^ of 0.998, and efficiency of 99.66%.

## DISCUSSION

Polymerase chain assays are commonly used, both quantitatively and qualitatively, for the diagnosis and evaluation of viral load in HIV-infected patients[15,16]. There are a number of methods, such as *in vitro* transcription, for producing positive RNA as an internal or external control in PCR assay[17]. RNA molecules are unstable in hard conditions such as high pH and high temperatures. In addition, RNA is easily degraded in the presence of divalent cations. Moreover, RNase is abundant in the environment; even minor contamination makes the RNA molecule extremely vulnerable[18]. Therefore, the synthesis of RNA and improvement of its stability are very important for the nucleic-acid-based assays. In the past decade, AR RNA technology has been developed to improve the stability of RNAs, used as standard or control in clinical diagnostic assays. Based on the use of AR RNA technology, target RNA was synthesized in *E. coli* by transformed plasmid and assembled along with the phage genome in the MS2 capsid; these PLPs contain the target sequence and are resistant to ribonuclease enzyme. The aim of current study is the constructing the positive-control RNA based on AR RNA technology, using HIV-1 RNA as a model.

To construct the positive-control AR RNA, 1751 bp of MS2 genome containing maturase, coat, and packaging sequence was cloned into pET32a plasmid. Moreover, 336 bp of *pol* conserved region of the HIV-1 genome was ligated downstream of MS2 gens in pET32a. The recombinant plasmid was transferred into *E. coli* strain BL2 (DE3), and the AR RNA was assembled following to IPTG induction. We also have shown the durability of AR RNA in the presence of ribonuclease enzyme. The sequence of coat gene that has an overlap region with lysis gene of the phage was mplified with PCR to prepare the plasmid that could produce large numbers of PLPs without bacterial lysis. Therefore, the produced PLPs were not infectious in accordance to biosafety concerns.

In a previous study, HIV-1 subtype B RNA was packaged in AR RNA[13,14]. However, the dominant HIV-1 subtypes in Iranian patients are CRF-AD35, B, C, and D[19-21]. In the present study, RNA sequence (336 bp of HIV-1 CRF-AD35) was encapsulated into the MS2 PLP using pET32a expression vector in *E. coli* DE3 (BL-21) strain.

The current PLP purification method involves the combined use of gradient ultracentrifugation, column chromatography, CO_2_ affinity chromatography, RNA-protein complex, and electroelution from an agarose gel with a GeBAflex-tube[22,23]. In the present study, PLPs were precipitated with PEG 6000 and further purified with Sephacryl S-200. The AR RNA was stable after treatment with DNase I and RNase A; this result is similar to other previous studies[14,24]. In another study, a part of the HIV-1 virus genome was packed in the MS2 phage capsid, and the PLPs were assembled based on AR RNA technology. Moreover, in a study by Burchard *et al*.[25], the cyber green dye was used to perform PCR reaction, but in the present study, we used the TaqMan method that is more specific than dsDNA-binding dye. Furthermore, in other studies[26], MS2 phage was used as an internal control for monitoring the cDNA synthesis steps in clinical specimen and nucleic acid preparation process, including lysis of the pathogen. In addition, the AR RNA was challenged by ribounuclease enzyme, and the stability of AR RNA was compared with naked RNA. The results of current study indicated that the stability of AR RNA in the presence of ribonuclease enzyme and durability of AR RNA and naked RNA was similar to previous studies[27,28]. In the current study, PLP standard was prepared and used as a positive control in real-time RT-PCR. The duplicate of real-time RT-PCR assay had high sensitivity, and the LoD of the channel (FAM) was 10^1^ copies/µl of AR RNA, indicating a constantly high amplification efficiency (>99%).

There are several commercial RNA controls for various infectious agents based on AR technology, but due to the high cost of these controls, their usage is limited in developed countries.

The results obtained in our study could be a basis for the stabilization of other infectious agents. Moreover, we have developed a virus-based control that requires an accurate analysis and evaluation with a panel of other infectious agents, as well as HIV-1 positives sample. This positive control can be used as a basis for launching an in-house HIV-1 virus assay and can be made it readily available to laboratories and HIV research centers.
